# Exposure as Part of a Systems Approach for Assessing Risk

**DOI:** 10.1289/ehp.0800407

**Published:** 2009-04-08

**Authors:** Linda S. Sheldon, Elaine A. Cohen Hubal

**Affiliations:** 1 National Exposure Research Laboratory and; 2 National Center for Computational Toxicology, U.S. Environmental Protection Agency, Research Triangle Park, North Carolina, USA

**Keywords:** computational biology, exposure science, modeling, risk assessment, systems biology

## Abstract

**Background:**

The U.S. Environmental Protection Agency is facing large challenges in managing environmental chemicals with increasingly complex requirements for assessing risk that push the limits of our current approaches. To address some of these challenges, the National Research Council (NRC) developed a new vision for toxicity testing. Although the report focused only on toxicity testing, it recognized that exposure science will play a crucial role in a new risk-based framework.

**Objective:**

In this commentary we expand on the important role of exposure science in a fully integrated system for risk assessment. We also elaborate on the exposure research needed to achieve this vision.

**Discussion:**

Exposure science, when applied in an integrated systems approach for risk assessment, can be used to inform and prioritize toxicity testing, describe risks, and verify the outcomes of testing. Exposure research in several areas will be needed to achieve the NRC vision. For example, models are needed to screen chemicals based on exposure. Exposure, dose–response, and biological pathway models must be developed and linked. Advanced computational approaches are required for dose reconstruction. Monitoring methods are needed that easily measure exposure, internal dose, susceptibility, and biological outcome. Finally, population monitoring studies are needed to interpret toxicity test results in terms of real-world risk.

**Conclusion:**

This commentary is a call for the exposure community to step up to the challenge by developing a predictive science with the knowledge and tools for moving into the 21st century.

The U.S. Environmental Protection Agency (EPA) and other regulatory agencies are responsible for managing large numbers of environmental chemicals. Although current regulatory decisions are based on a wide range of tools and information that represent the best available science, often limited or no exposure or toxicity data are available for making these decisions (Judson et al. 2009). Recent statutory changes require increasingly complex approaches for evaluating the impact of life-stage vulnerability, genetic susceptibility, varying exposure scenarios, and exposures to multiple stressors on environmental health risks. These new requirements push the limits of our current tools and scientific understanding. Fortunately, the rapid explosion of new computational, physical, and biological science tools have the potential to address these challenges and to transform the ways in which exposure and toxicity testing come together to assess health risks.

Because of the number of chemicals involved and the increasing complexity of future assessments, new approaches are needed. To examine and address these limitations, the National Research Council (NRC) evaluated the issues and developed a framework for toxicity testing as it could be applied to risk assessment. The report, *Toxicity Testing in the 21st Century: A Vision and Strategy* (NRC 2007), articulates a long-range vision that applies systems biology, rapid assay technologies, and bioinformatic tools to improve toxicity testing. Although the focus was intentionally on toxicity testing, the NRC recognized that exposure must be a key component if the intended goal is to evaluate risks and inform public health decisions. Exposure science, when incorporated throughout the entire framework, will increase the efficiency of the testing process, help inform toxicity testing, describe risks, and verify the outcomes of new risk assessment approaches. The NRC recommended that exposure science be considered at every step in the new testing and risk assessment strategy.

In this commentary, we establish a strategic framework for the exposure research needed to achieve a new approach for risk assessment. Crucial to this vision is the application of a systems approach that fully integrates exposure and toxicity information in a holistic framework for improved public health decision making. We also elaborate on the exposure research needed to achieve this new vision.

## A Systems Approach for Assessing Risk

The authors of *Toxicity Testing in the 21st Century* (NRC 2007) proposed to use systems biology to serve as the basis for a new toxicity-testing paradigm. The fundamental construct is to develop *in vitro* tests to characterize toxicity pathway perturbations and then predict health impacts that could result from these perturbations. If we broaden this vision, systems theory also provides the required conceptual framework for linking exposure science and toxicology in order to study, characterize, and predict the complex interactions between humans and environmental chemicals that lead to health risks.

Toxicity pathways, as articulated by the NRC, are normal pathways for maintaining cellular functions that, when sufficiently perturbed, will lead to an adverse health outcome. The consequences of a perturbation depend on its magnitude, which is related to dose at the cellular level, the timing and duration of the perturbation, and the susceptibility and life stage of the host. Exposure science provides information on the magnitude, timing, and duration of individual exposure as well as the resulting dose at the tissue, cellular, and even molecular level (Cohen Hubal et al. 2008). Importantly, exposure information will determine whether toxicity pathways can be perturbed and whether there is a risk.

A fully integrated systems approach will reduce many of the uncertainties with current risk assessment approaches. Understanding the mechanisms of toxicity pathways will reduce uncertainties associated with using animal data to predict human risk. When integrated with exposure and dose information, it also affords the opportunity to reduce uncertainties associated with using high doses to predict risk at lower environmental exposures, predict cumulative risks, and predict risk to susceptible populations.

## Exposure Science for the 21st Century

Because of the complex nature of the human system, health risk predictions associated with chemical exposures will be only as good as the least resolved or least understood component. Advanced tools are available to rapidly examine toxicity pathways at a depth and breadth not previously possible. For a fully integrated system, a comparable set of advanced exposure tools must be developed; these tools must be rapid, efficient, and predictive.

Exposure science provides the linkages between what is present in the environment and the internal dose that individuals and populations receive. A strategic long-term program for exposure research must develop predictive computation tools based on a mechanistic understanding of important (i.e., rate-limiting) exposure processes and determinants. High-priority research needs include the development and application of

- integrated modeling approaches to reliably predict exposure and dose
- highly efficient screening tools for chemical prioritization
- easily accessible exposure databases aligned with toxicity databases
- efficient and affordable tools for generating new exposure and dose data.

### Integrated modeling approaches for predicting exposure and dose

Computational models that can be efficiently integrated to predict exposure and dose at the toxicity pathway are fundamental to the new risk assessment vision. These models, in turn, should be integrated with dose–response and biological pathways models to describe the entire source to outcome continuum.

**Figure 1:**
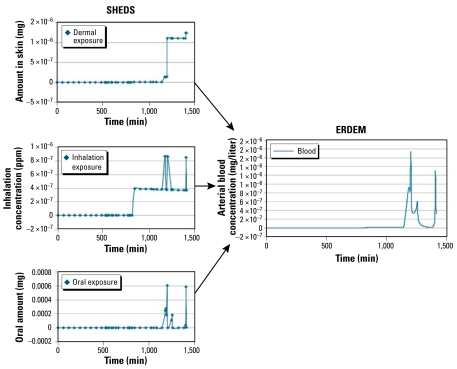
Illustration of linked exposure (SHEDS) and dose (ERDEM) models for methyl *tert*-butyl ether.

Exposure models estimate concentrations of chemicals in environmental media and describe activities that bring individuals into contact with the contaminated media. Several models have been developed and applied for this purpose (Williams et al., in press). The U.S. EPA Stochastic Human Exposure and Dose Simulation (SHEDS) model (U.S. EPA 2009a) can track activities minute by minute throughout the day and link these activities to environmental concentrations to estimate exposures by specific route and pathways (Zartarian et al. 2006). The longitudinal aspect of the model provides the ability to estimate not only the magnitude, but also the frequency and duration of exposure over the same time period. When SHEDS model outputs are linked to a physiologically based pharmacokinetic (PBPK) model such as U.S. EPA’s Exposure Related Dose Estimating Model (ERDEM) (U.S. EPA 2007; Zhang et al. 2007), the magnitude, frequency, and duration of internal dose can also be predicted. Figure 1 illustrates this linkage for methyl *tert*-butyl ether exposures.

Integrated exposure/PBPK models can be used in several ways. Outputs can be used directly to inform toxicity testing as well as to conduct quantitative risk assessments. Linked models can simulate dose for multiple routes (inhalation, ingestion, and dermal) and multiple chemicals simultaneously, thus providing the ability to evaluate cumulative risks. Integrated models also provide the ability to evaluate risks to susceptible populations by considering differential activities that could change exposures or differential physiology that could affect adsorption, distribution, metabolism, or elimination characteristics. Finally, the models can be used in reverse for dose reconstruction as an alternative approach for comparing toxicity testing results to population exposures (Georgopoulos et al. 2008; Tan et al. 2007).

Realizing the potential for integrated modeling approaches requires a coordinated and sustained research effort. PBPK models need to be extended to allow dose estimation at the cellular and molecular level. Integrated exposure/PBPK models must be enhanced to provide distributional outputs along with uncertainty and variability. Developing systems that are efficient and generalizable must be a part of this effort. New data and new approaches are needed for exposure reconstruction in order to reduce uncertainties with current approaches. Current efforts (Georgopoulos and Lioy 2006; Rosenbaum et al. 2007) to provide models that use a common platform and/or common programming language must continue.

At the same time, research is required to develop approaches for estimating model inputs and parameters without resource-intensive and burdensome studies. For example, environmental informatics, quantitative structure–activity relationships (QSARs), and computational chemistry approaches should be developed to predict and quantify behaviors such as environmental fate and transport or metabolism. Development of metabolic predictors or simulators that can address single chemicals, multiple chemicals, and the interaction among chemicals should be accelerated (Mekenyan et al. 2005). Novel statistical and informatic approaches should be applied to extant exposure data to facilitate the identification of critical metrics that represent personal exposure through time, place, life stage, lifestyle, or behavior.

### Exposure screening tools for accelerated chemical prioritization

Current risk assessment approaches cannot meet demands for the large number of chemicals that must be evaluated. Screening tools are needed that reliably identify those chemicals that will require more comprehensive risk assessments. Chemical prioritization should consider both exposure and hazard. The U.S. EPA, through its ToxCast program (U.S. EPA 2009b), is developing rapid *in vitro* assays to screen chemicals for further testing based on toxicity (Dix et al. 2007). Innovative rapid-screening tools based on exposure are also needed. Predictive approaches for estimating important parameters for screening need to be developed. Ideally, these tools should account for chemical use, physical and chemical properties, occurrence and co-occurrence of chemicals, potential exposure scenarios, routes of exposure, and various exposure factors. This will include developing approaches that describe a chemical’s behavior in the environment as well as approaches to identify important human activities that will impact exposure. Exposure prioritization approaches will require easily accessible databases, as described below.

One plausible approach may be to formulate an exposure classification index based on a limited set of metrics designed to efficiently cover exposure potential (Cohen Hubal et al. 2008). As a first step, innovative approaches for chemical prioritization (e.g., Arnot and MacKay 2008; Hays et al. 2007) as well as indexing approaches from other fields should be reviewed and mined. This index could be “trained” on data-rich chemicals and products and then validated on a representative set of chemicals for which little exposure data are available. In this way, a limited set of critical metrics could be identified for efficient screening of new chemicals. Finally, because consumer products often incorporate multiple chemicals in a variety of forms, rapid experimental screening protocols that measure the potential for availability or release of these compounds into exposure media are under early development and should be pursued further (Little and Cohen Hubal, in press).

Significant research and model development activities are currently under development within the U.S. EPA (U.S. EPA 2005) as well as in Canada and Europe (Bridges 2007; Environment Canada 1999; Van der Wielen 2007). Partnerships with these groups should be fostered to leverage and establish collaborative exposure science research for future chemical screening and prioritization.

### Exposure databases

Easily accessible exposure databases that can be linked to each other and with toxicity databases can and should be developed immediately. Data on chemical manufacture, product use, environmental fate, media concentrations, biomarker levels, and metabolism should be identified. International standards for exposure data representation should be discussed. Approaches for improving access to human exposure data and for facilitating links between exposure and toxicity data should be implemented. Existing tools and platforms that are currently being implemented with environmental toxicity information should be adapted for exposure information to provide the most useful links to existing toxicity data. Chemical structure annotation of exposure-related data, such as could be provided by the Distributed Structure Searchable Toxicity (DSSTox) database (Richard et al. 2006, 2008; U.S. EPA 2009c), and incorporation of such data into the new Aggregate Computational Toxicology Resource (ACToR) (Judson et al. 2008) will greatly enhance linkages between these data and toxicity-related human health end points. For maximum impact, this activity should be conducted in collaboration with international partners working to achieve similar goals (Environment Canada 1999; Van der Wielen 2007).

### Efficient monitoring methods for assessing risk

Population-based and surveillance studies will provide the ability to link the results from toxicity testing to the real world and to track our progress in protecting public health. To be feasible, new low-cost, low-burden methods and approaches for conducting these studies will be needed. New technologies need to be applied to develop a toolbox of methods for assessing exposure, susceptibility, and biological response in large surveillance studies. New sensor technologies, applications of nano-technology, geographic information systems, and genomics assays need to be developed and put into use for this purpose.

Emerging tools in molecular biology provide the potential to develop cellular and molecular indicators of exposure and biological response. Better understanding of genomic expression may also provide insight into factors impacting differences in susceptibility to chemical exposure in the human population (Oberemm et al. 2005). “Omics” should be explored as a way to identify expression patterns associated with exposure to individual chemicals or chemical mixtures. Such technologies could then provide linkages between exposure and health outcomes in population studies. Development of environmental and/or molecular indicators of exposure combined with development of novel sensor-based monitoring tools will present the opportunity for simultaneous, near-real-time measurement of exposure and dose to multiple real-world stressors in mixtures (Weis et al. 2005). A strong and immediate research effort is required for novel technologies that will generate the data required for risk assessments and decisions that truly protect public health.

## Conclusions

Exposure science is crucial for addressing many of our important and complex environmental health issues. As discussed here, exposure science is essential for toxicity testing to be valuable in public health protection. A systems approach is required that fully integrates exposure and toxicity into a holistic framework for risk assessment.

The exposure community must step up to the challenge to develop a robust and predictive science that can be used to address the complex problems in the 21st century. A research program that provides the necessary exposure data and tools within an integrated framework will need to be multidisciplinary and take advantage of collaborative opportunities. Key to this work are strong collaborations within the exposure community and with those researchers who are developing information on toxicity pathways and conducting toxicity testing. Multiple collaborations are needed to ensure that

- chemical prioritization considers both exposure and toxicity
- databases are developed
- analysis is conducted using these databases to understand exposures, doses, and toxicity
- new information on biological interactions and pathways is used to develop the appropriate indicators for exposure and surveillance studies
- models on exposure and dose are linked for extrapolations
- feedback loops are developed to inform future planned research.

Collaborations should include partnerships between governmental and nongovernmental research groups as well as academia and industry for developing and applying new exposure tools. Just as a new vision and initiatives have been developed for toxicity testing, it is now time for the exposure community to dedicate itself to engaging in similar activities to move our science into the 21st century.
